# A CNN Hyperparameters Optimization Based on Particle Swarm Optimization for Mammography Breast Cancer Classification

**DOI:** 10.3390/jimaging10020030

**Published:** 2024-01-24

**Authors:** Khadija Aguerchi, Younes Jabrane, Maryam Habba, Amir Hajjam El Hassani

**Affiliations:** 1 MSC Laboratory, Cadi Ayyad University, Marrakech 40000, Morocco; khadija.aguerchi@edu.uca.ma; 2 National School of Applied Sciences of Safi, Cadi Ayyad University, Safi 46000, Morocco; m.habba@uca.ma; 3 Nanomedicine Imagery & Therapeutics Laboratory, EA4662—Bourgogne-Franche-Comté University, 90010 Belfort, France; amir.hajjam-el-hassani@utbm.fr

**Keywords:** breast cancer, deep learning, image classification, Convolutional Neural Networks, Particle Swarm Optimization

## Abstract

Breast cancer is considered one of the most-common types of cancers among females in the world, with a high mortality rate. Medical imaging is still one of the most-reliable tools to detect breast cancer. Unfortunately, manual image detection takes much time. This paper proposes a new deep learning method based on Convolutional Neural Networks (CNNs). Convolutional Neural Networks are widely used for image classification. However, the determination process for accurate hyperparameters and architectures is still a challenging task. In this work, a highly accurate CNN model to detect breast cancer by mammography was developed. The proposed method is based on the Particle Swarm Optimization (PSO) algorithm in order to look for suitable hyperparameters and the architecture for the CNN model. The CNN model using PSO achieved success rates of 98.23% and 97.98% on the DDSM and MIAS datasets, respectively. The experimental results proved that the proposed CNN model gave the best accuracy values in comparison with other studies in the field. As a result, CNN models for mammography classification can now be created automatically. The proposed method can be considered as a powerful technique for breast cancer prediction.

## 1. Introduction

Each year, approximately half a million women worldwide die of breast cancer [1]. This disease can be fatal if it is not detected early. Detection of breast cancer using mammography has been utilized over the last three decades in numerous nations throughout the world to reduce the breast cancer death rates. The combination of screening and advances in treatment has reduced breast cancer mortality by 30% [1]. Death rates have gone down thanks to improvements in mammography screening and traditional computer-aided diagnostic (CAD) models [2,3]. Currently, CNNs are used for a variety of tasks due to their considerable performance. CNNs can automatically be trained using training data, which has an advantage over conventional feature representation. Recently, the performance of deep learning and Convolutional Neural Network (CNN) algorithms in image classification has significantly improved, as has the detection of lesions by mammography and the evaluation of image quality [4]. A variety of methodologies have been used to increase the accuracy of deep CNNs [5,6].

Designing a real-world application using CNNs remains challenging due to the difficulty of selecting the optimum parameters [7]. Therefore, optimization methods are used to adjust the model’s hyperparameters. In the literature, many optimization algorithms have been presented for various industrial applications, such as Particle Swarm Optimization (PSO) [8]. The hyperparameter values employed in a deep learning model significantly affect its performance. The objective of hyperparameter optimization is to look for the optimal hyperparameter settings. Since the search area is huge and evaluating every setting can be costly, hyperparameter optimization is often performed manually.

This paper aimed to solve the problem of finding CNN architectures that achieve a high classification accuracy for image classification. This is why we present our implementation of PSO, called PSOCNN, to address such a problem. PSOCNN focuses on fine-tuning the CNN’s hyperparameters, which can control the design of the CNN and, therefore, impact its classification accuracy. The main contributions we made are as follows:

1. This study performed, for the first time, the optimization of new hyperparameters (kernel size, stride, filter number) for CNNs used for mammography image classification.
2. Optimal architectures for deep Convolutional Neural Networks were found using a unique PSO technique.
3. We developed an improved algorithm for finding relevant CNN designs compared to existing methods.
4. Compared to default or random settings, the proposed approach reduces the manual effort required to determine the appropriate configuration for a CNN to achieve peak performance. The manual search for such dataset-specific setups necessitates much human time and knowledge. As a result, the proposed approach will encourage non-experts to efficiently employ neural network architectures.

This article is divided into three sections. Following the Abstract, an introduction and review of the related works are detailed in Section 1. Section 2 presents the materials and methods. Section 3 covers the experiments and results. In Section 4, this study is analyzed and conclusions are drawn, as well as some recommendations for future research.

### Related Works

From previous research, several studies have been performed to develop a deep CNN architectures for the classification of both natural and medical images. A few deep-learning-based methods for classifying breast cancer masses in mammography pictures have been proposed. In [9], authors created a deep-belief-network (DBN)-based approach for determining whether mammography images are normal or abnormal. A discrete wavelet transform was used to extract image features, specifically the gray level co-occurrence matrix features from the HL and LL wavelet sub-bands. The authors showed that a deep belief network (DBN) CAD system could enable automated hierarchical feature extraction to offer more flexibility for intricate design patterns. Yet, there are several obstacles to overcome in this approach, including the demand for large and varied datasets for efficient performance, the need for extensive computer resources during training, and possible issues understanding the learned features. Moreover, to successfully build a DBN-based CAD system, it is imperative to strike a balance between model complexity and resource requirements. The results on the MIAS dataset included an accuracy of 91.5%, a specificity of 72.4%, and a sensitivity of 94.1%. The authors in [10] proposed a CNN approach for automatically classifying breast cancer from mammography and ultrasound images. The method has five adjustable convolutional blocks, each composed of four convolutional layers, with a single fully connected layer, which serves as the classifier. The method uses a few customizable parameters to automatically extract key features from images. The benefit of using deep Convolutional Neural Networks (CNNs) and a multi-modal approach is that they allow for an automated diagnosis and thorough analysis. Nonetheless, some difficulties can occur due to the CNN model’s interpretability, its reliance on large and high-quality data, the requirement of strong validation, and moral issues surrounding healthcare automation. The authors performed many simulations on mammography datasets (DDSM, MIAS, and INbreast) and ultrasound datasets (BUS-1 and BUS-2) and found that their evaluation metrics were better than the current best practices. Furthermore, data augmentation allowed for less overfitting. Using the DDSM, MIAS, and INbreast datasets, their CNN algorithms obtained accuracies of 90.68%, 96.55%, and 91.28%, respectively. Additional accuracies of 100% and 89.73% were achieved on the BUS-1 and BUS-2 datasets, respectively. In [11], the authors began by removing noise, then adding a logarithmic spatial transform to improve the images, and finally, deleting the oblique and pectoral muscles and backdrop. Then, they used a fractional Fourier transform to obtain the coefficients of the time–frequency spectrum, which were then reduced using the PCA technique. In conclusion, the following performance results were obtained using the classifiers (k-nearest neighbors and SVM): in the case of SVM, the sensitivity was 92.22%, the specificity was 92.10%, and the accuracy was 92.16%. In addition, in [12], the researchers suggested the use of a multi-scale all-Convolutional Neural Network (MA-CNN) for the classification of mammography images. To keep the connections between close pixels, instead of pooling, a long stride convolution was used. The possible advantages include the multi-scale approach and the particular emphasis on mammography characteristics. This notwithstanding, there are some limitations to this method, such as its dependence on varied and high-quality data, its interpretability, the necessity of a thorough validation, possible computing demands, and its practical application in a clinical environment. The obtained sensitivity, accuracy, and AUC on the MIAS dataset were 96%, 96.47%, and 99%, respectively.

In [13], a YOLO-based CAD system utilizing deep learning was introduced to detect and classify masses related to breast cancer. The methodology comprises four sequential steps: After completing preprocessing, the model uses a deep convolutional network to extract the features. Mass detection is then performed, and a fully connected neural network is used for mass classification. The Digital Database for Screening Mammography (DDSM) and a pre-trained model from the ImageNet dataset were utilized, with weights assigned accordingly. Finally, the model was fine-tuned. The YOLO technique was employed in computer-aided design (CAD) to detect objects in real-time precisely. This enables the efficient processing of large CAD files. Its versatility and effectiveness make it possible to identify a wide range of object classes and integrate them into current systems. When it comes to localizing small objects, the YOLO method may not be as accurate as slower options. In CAD settings with limited resources, the large and complicated models and the need for many training data can be a problem. The effectiveness of YOLO depends on the needs of the particular CAD application, and the tradeoff between speed and accuracy is a factor. With the help of two different datasets from the DDSM database, consisting of an original amount of 600 photos and their augmented set of 2400 images, the researchers evaluated their system’s performance. They achieved an impressive overall accuracy of 97% and an area under the curve (AUC) of 96.45%. The researchers in [14] presented a deep-belief-network (DBN)-based CAD system for identifying breast cancer. This approach involves the extraction of regions of interest (ROIs) utilizing two distinct methodologies. The initial approach selects four randomly chosen regions of interest (ROIs) from an identified mass, with each ROI having dimensions of 32 by 32 px. The second technique makes use of every ROI that has been found. This technique employs morphological processes and adaptive thresholding to detect masses with an accuracy of 86%. Due to its limitations in detecting masses in dense regions, many forms of breast tissue pose difficulties in identification and diagnosis. After the extraction of the ROIs, this technique provides 347 statistical parameter settings to identify the optimal one. Most of the time, hyperparameter tuning is performed by hand because the search area is large and it can be costly to test each setup. They then used deep belief networks (DBNs) to classify the images. The classification technique achieved an accuracy of 90.86% for malignant tumors and 92.86% for benign tumors. Additionally, the AUCs for the total mass method and the ROI mass method were 93.54% and 86.56%, respectively. A technique for identifying and classifying breast cancer using mammogram images was presented in [15]. The researchers used a Convolutional Neural Network to classify mammogram images after several preprocessing procedures to adjust the CNN classifier’s parameters. Using the MIAS dataset, they achieved a percentage of 82.71% for the accuracy. The authors in [16] developed a classification system for mammogram images called CNN improvement for breast cancer classification (CNNI-BCC). The CNNI-BCC model classifies the images of breasts into three classes: malignant, benign, and normal masses. They had an accuracy rate of 90.50% and a specificity of 90.71%. Although evolutionary algorithms have been employed to optimize CNN parameter values, their use has not received much research. Today, in research, metaheuristic algorithms are employed for designing deep learning architecture. For example, the authors in [17] used both fuzzy logic modeling and a better quantum-behaved Particle Swarm Optimization method. To evaluate the impact of particular fuzzy variables on surface degradation, they conducted ball-on-disk tests. They improved the fuzzy model by making the fuzzy variables’ membership functions more optimal to increase the prediction accuracy. In [18], the researchers developed a novel method to detect breast cancer in mammogram images, leveraging feature extraction and reduction techniques. The authors utilized various pre-trained Convolutional Neural Network (CNN) models to extract the features. These features were then combined, and the most-useful ones were chosen based on mutual information. The selected features were then classified using various machine learning algorithms, including neural networks (NNs), k-nearest neighbors (kNN), random forest (RF), and support vector machine (SVM). The proposed algorithm was evaluated on different datasets, including the newly introduced RSNA dataset, MIAS, and DDSM. The authors in [19] conducted research to assess the effectiveness of genetic algorithms in the context of neural network classifiers for categorizing land cover in multispectral remote sensing data. A genetic algorithm analysis was performed in a hybrid environment with backpropagation, but the network properties and how they affect categorization were not specifically thought about.

Lorenzo et al. utilized Particle Swarm Optimization (PSO) to select the deepening parameters, as described in their study [20]. The algorithm is based on natural behavior and imitates flocks of birds or fish. It was initially proposed by Eberhart and Kennedy in their publication [21]. Based on the experiment results using the LeNet-4 network, it was shown that PSO can significantly improve the accuracy of classification on the MNIST dataset. However, their approach and methodology were not suitable for the CIFAR-10 datasets.

To be more precise, the large number of hyperparameters is an obstacle to achieving better results, despite the positive findings reached by CNN architectures in the detection of breast cancer. As a result, optimizing the hyperparameters for CNN design is crucial to enhancing CNN performance. This study developed an enhanced Convolutional Neural Network (CNN) structure for classifying mammography datasets. The architecture was refined using the Particle Swarm Optimization (PSO) approach to determine new hyperparameters. This might potentially be advantageous for healthcare professionals in the diagnosis of breast cancer.

## 2. Materials and Methods

### 2.1. Datasets

Two different standard mammography image datasets were used to create and assess the proposed algorithm. These datasets, the Digital Database for Screening Mammography (DDSM) and the Mammographic Image Analysis Society (MIAS), were already preprocessed in [22]. Mammography images containing both benign and malignant masses are included in this dataset. The authors of [22] used three original sets of images from other datasets to make this dataset: 106 masses from INbreast, 53 masses from MIAS [23], and 2188 masses from DDSM [24]. DDSM comprises both normal and abnormal examples, making it ideal for examining the performance of computer-aided detection (CAD) systems. Masses and microcalcifications are examples of abnormalities. DDSM has been widely employed in the creation and testing of computer-aided diagnosis (CADx) systems, image-processing methods, and other breast cancer detection applications.

MIAS is best suited for tasks involving breast cancer detection since it concentrates on instances with worrisome lesions. Digitized film mammograms are included in the database.

MIAS, like DDSM, has annotations that indicate the presence of anomalies such as masses and calcifications. These annotations are critical for algorithm training and assessment.

**Figure 1:**
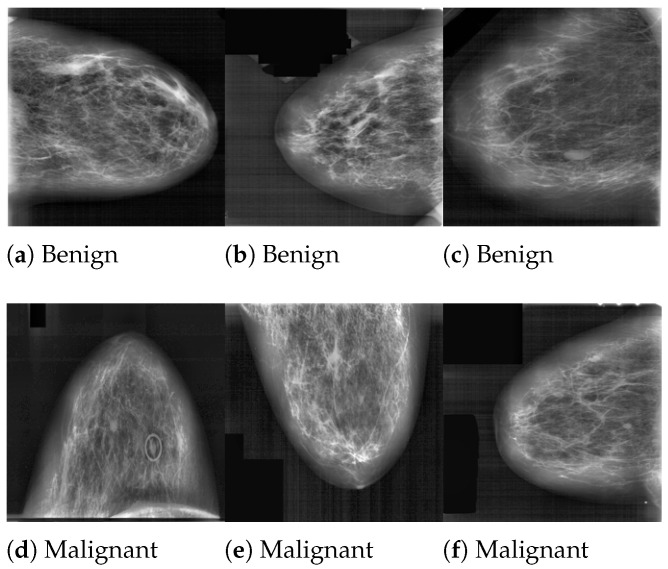
Samples of breast mammography from DDSM dataset [22].

**Figure 2:**
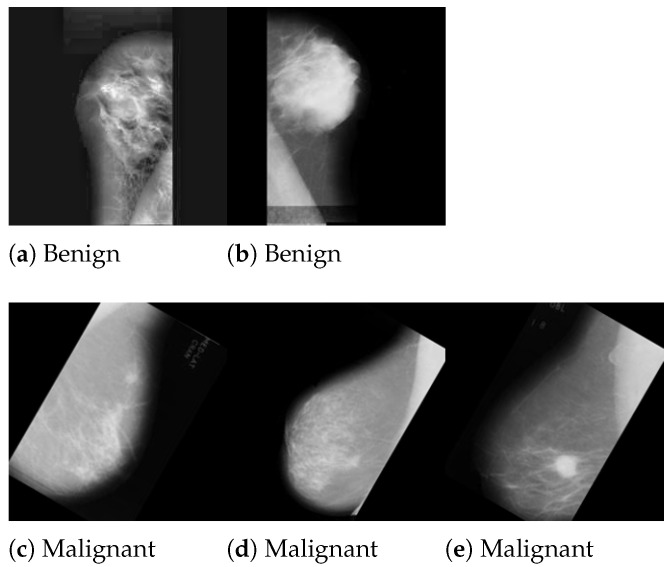
Samples of breast mammography from MIAS dataset [22].

After the researchers preprocessed their images, they next employed data augmentation and contrast-limited adaptive histogram equalization. Following data augmentation, the INbreast dataset has 7632 pictures, the MIAS dataset has 3816 images, and the DDSM dataset has 13,128 images. In addition to that, they combined DDSM, MIAS, and INbreast. The new size for each picture was 227 × 227 px. In this experiment, the DDSM and MIAS datasets were employed. Figure 1 and Figure 2 present the samples of the DDSM and MIAS datasets used in this study. Table 1 shows the details of the entire dataset.

### 2.2. CNN Hyperparameters’ Optimization

The optimization of Convolutional Neural Networks’ parameters involves determining the suitable parameters that result in significant accuracy for each task. Nevertheless, the task of enhancing a large number of parameters is extremely difficult, with a high computational cost. Therefore, it is necessary to implement optimization algorithms that lead to a reduced number of iterations. The present study was based on the Particle Swarm Optimization (PSO) technique to look for the CNN model with the highest accuracy for breast cancer detection. Developing a Convolutional Neural Network (CNN) involves the optimization of several parameters and the careful selection of the architecture. The selection of optimal parameters is crucial for obtaining accurate results when using Convolutional Neural Networks (CNNs). Therefore, it is a challenging task that requires a considerable level of expertise.

The effectiveness of a CNN model depends on its hyperparameters, therefore driving certain researchers to advocate for the indispensability of fine-tuning these hyperparameters to obtain positive results. Hence, it is a challenging task that requires a substantial degree of proficiency. The hyperparameters of the CNN architecture, together with their descriptions, are provided in Table 2. As previously mentioned in Section 1, metaheuristic algorithms are widely acknowledged as effective techniques for enhancing the performance of CNN architectures by optimizing their hyperparameters.

### 2.3. Particle Swarm Optimization

The Particle Swarm Optimization (PSO) approach is widely used as a metaheuristic tool for solving discrete, continuous, and combinatorial optimization problems. In 2001, Kennedy and Eberhart created the original version [25]. It took its cue from a flock of birds’ flight pattern. A single solution is referred to as a particle in PSO, while the collection of all solutions is referred to as a swarm. Particles and swarms are terms used in the context of PSO to refer to both individual solutions and collective ones. PSO’s fundamental tenet is that each particle only knows its current velocity, its best configuration to date (pBest), and the particle in the swarm that is now the best in the world (gBest). In every iteration, every particle modifies its velocity to obtain its new location closer to both its pBest and gBest. The equation below modifies each particle’s velocity v:

(1) $$v_{i,j}\left(t+1\right)=w\times v_{i,j}\left(t\right)+c_{p}\times r_{p}\times \left(gBest_{i,j}-x_{i,j}\left(t\right)\right)+c_{g}\times r_{g}\times \left(gBest_{j}-x_{i,j}\left(t\right)\right)$$

where *v_i,j_* is the particle’s velocity in the *j*-th dimension, *x* is the particle’s current location, and *w* is a constant called momentum that regulates how much the velocity from the previous time step will influence the velocity at the present step. *c_p_* and *c_g_* are predefined constants, whereas *r_p_* and *r_g_* are random values in the range [0, 1]. Additionally, by changing the variables *c_p_* and *c_g_*, respectively, the algorithm’s capacity for exploration and exploitation may be adjusted. Finally, the following changes are made to the *i*-th particle’s location in the *j*-th dimension:

(2) $$x_{i,j}\left(t+1\right)=x_{i,j}\left(t\right)+v_{i,j}\left(t+1\right)$$

The PSO algorithm’s major steps are:

1. Initialize the population values of the particles.
2. Determine the population’s fitness.
3. Recall the best solution.
4. Repetition:
  (a) Each particle’s position and velocity should be updated in line with Equations (1) and (2).
  (b) Determine the fitness value of every particle in the population.
  (c) Refresh the best solution.
5. Continue until a final criterion is met.

**Figure 3:**
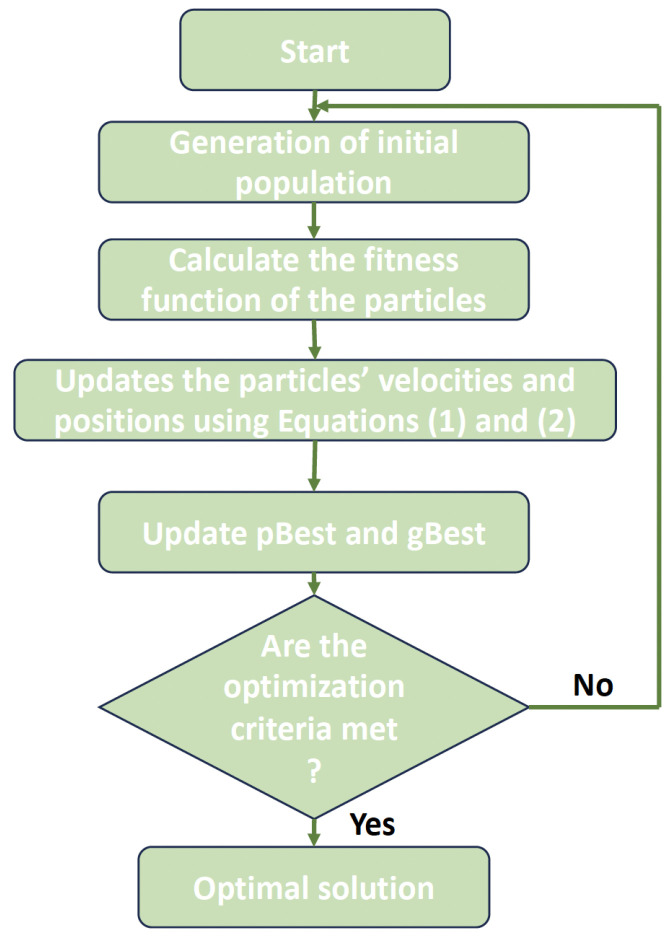
The basic process of PSO.

The PSO process is shown in Figure 3.

One of the most-advantageous features of PSO is that it converges quicker than GAs [26,27]. Because a single CNN training run might take many days even on the most-powerful computers, this property can be advantageous when looking for optimum CNN structures.

Due to the large number of parameters in even simple CNNs, the training procedure can only be accomplished with graphic processing units (GPUs). The duration of a single training run can vary from days to weeks, depending on the complexity of the CNN architecture. Consequently, experimenting with many CNN designs through trial and error can be extremely time consuming. As a result, it is critical to create algorithms capable of autonomously building and assessing CNN designs as quickly as is feasible.

### 2.4. Proposed Method

#### 2.4.1. CNN Architecture Design

We built our own CNN in this part to train the Convolutional Neural Network (CNN) from scratch (a new model). It consists of three convolutional layers with three max-pooling layers, one dropout, a flattening layer, and two FC layers. The activation function for each layer is the ReLU function, except for the last one for the output, which is the Sigmoid function. The output layer uses a Sigmoid function, which maps the output value to the range of [0, 1].

#### 2.4.2. The PSOCNN Algorithm Overview

Using the PSO algorithm, which is defined in Figure 3, the proposed PSOCNN approach creates CNN structures. The detailed processes of the implementation of the PSOCNN algorithm to optimize the CNN architecture are shown in Algorithm 1 and summarized as follows:

First, specify all of the algorithm’s input parameters that are relevant to the problem at hand, such as the dataset that is needed for training and the settings for the CNN structures that need to be created.

Second, initialize the population. The Popsize particles are given during this phase. Each particle has several hyperparameters, and the particle positions, best personal position, and best global position are all initialized.

Third, at each iteration, each particle changes its position based on its own best position in the search space, called pBest, and the best position in the entire population, called gBest, using the update approach provided in Section 2.3. The procedure is continued, assessing all particles until the stopping criterion is met (in this paper, the number of iterations).

Each particle is compensated in a CNN architecture for evaluating the particles, trained, and tested against the dataset. The classification accuracy attained is then saved as the particle’s fitness. Then, update the best personal position (pBest) and the best global position (gBest).

If the maximum number of iterations is achieved, gBest is the best answer for our algorithm. Otherwise, we return to the third stage. This algorithm determines the optimal solution, which is the particle represented by gBest, which is the best CNN architecture for this dataset.

#### 2.4.3. The PSOCNN Process

This section outlines an optimization method that uses the Particle Swarm Optimization (PSO) metaheuristic algorithm to find the most-suitable parameters for the Convolutional Neural Network (CNN) architecture. The main objective is to determine the crucial parameters necessary for obtaining optimal performance in CNNs and, thereafter, employ the PSO methodology to attain these desired values. The following parameters were chosen for optimization in this work:

- Kernel Size (ks)
- Stride (s)
- Filter number (convolution layer) (nf)

The particles were created in this procedure by initializing the PSO by the execution parameters (detailed below). Every particle is a possible solution, and because every position contains a parameter that may be tuned, every solution is a finished CNN training.

**Figure 4:**
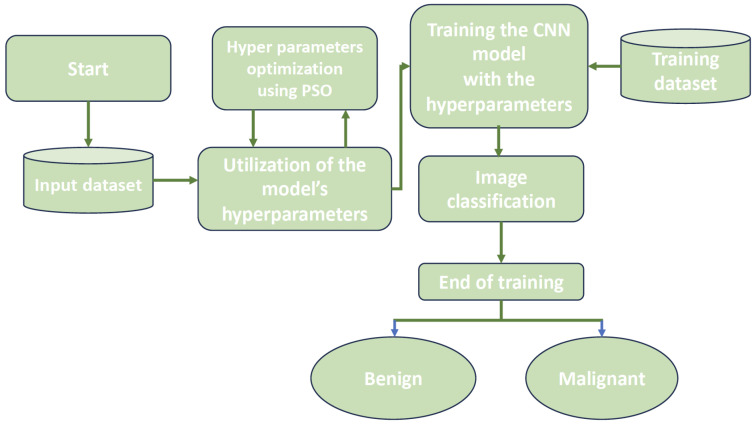
The process of the proposed system for classifying benign from malignant tumors.

**Figure 5:**
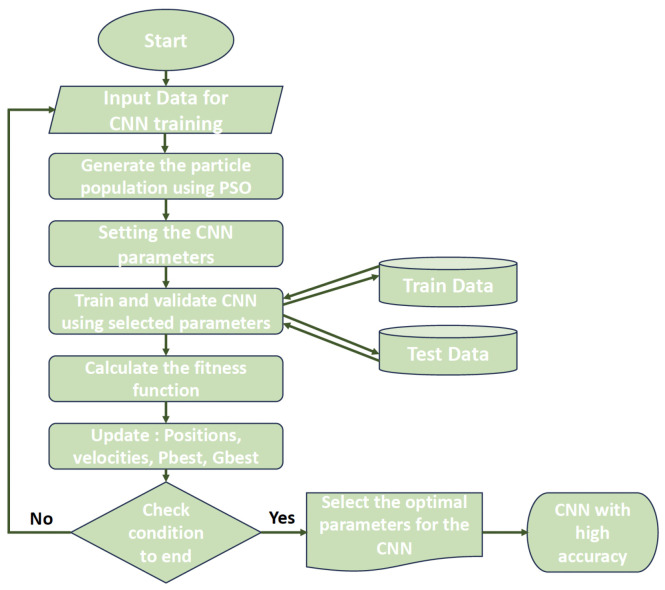
Flowchart illustrating how CNN is improved using PSO.

The procedures for optimizing the hyperparameters of the Convolutional Neural Network (CNN) using the Particle Swarm Optimization (PSO) approach are illustrated in Figure 4 and explained in detail in Figure 5:**Algorithm 1:** The proposed PSOCNN model.1:Initialize PSO parameters (population size, maximum iterations, etc.)2:Initialize particles randomly within the search space3:Initialize particle velocities randomly4:Initialize best particle positions and fitness values5:**while** Not converged and maximum iterations not reached **do**6: **for** each particle in the population **do**7:  Update particle velocity using PSO equations8:  Update particle position9:  Evaluate the fitness of the particle using the CNN10:  **if** Current fitness is better than personal best **then**11:   Update personal best position and fitness12:  **end if**13:  **if** Current fitness is better than global best **then**14:   Update global best position and fitness15:  **end if**16: **end for**17:**end while**18:Use the global best position as the optimized CNN architecture19:Train the CNN with the optimized architecture on the dataset20:Test the trained CNN on a test dataset

1. This work selected the DDSM and MIAS datasets for analysis and classification.
2. Generate the particle population for the Particle Swarm Optimization (PSO) method. The experiment’s parameters are listed in Table 3. The components of Particle Swarm Optimization (PSO) include particles, the number of iterations, the inertial weight (w), the social constant (c2), and the cognitive constant (c1).
3. Initialize the architecture of the CNN. Using the PSO parameters (filter number, filter size (convolution layer), and stride), the CNN hyperparameters are initialized, and they use other parameters listed in Table 4. The CNN is now prepared to train on the input images.
4. Model training and validation: The Convolutional Neural Network (CNN) processes and analyses the input data while splitting the images into training, validation, and testing sets. The accuracy of the model is evaluated during the testing phase. The PSO receives these values as a part of the objective function.
5. Examine the objective function: To obtain the ideal value, the PSO method examines the objective function.
6. Update the PSO parameters: Each velocity and position of the particle is updated during each iteration based on its particle’s best position (pBest) in the search range and the global best position of the entire swarm (gBestp).
7. The process is iterated, analyzing all particles until the stop conditions are met (the iteration number).
8. In the end, the best solution is chosen. The best particle for the CNN model is the one that gBestp represents in this procedure.

## 3. Experiments and Results

### 3.1. Experimental Studies

This section presents the training and test results for the CNN models created with PSO. The obtained values were compared with previous studies in the literature. Additionally, the initial parameter values used in PSO are also given, as are the values of the parameters used to create the CNN model.

The Python programming language was used in our tests. We ran our trials on Kaggle, which was used for the implementation, connected to Python 3 with a maximum of 30 GB of RAM, a 70 GB disk, and a CPU.

#### Parameters Involved in the Experiment

Some examples of static parameters in CNN parameter designs are the learning function, the classifying layer activation function, the nonlinearity activation function, and the number of epochs. The PSO configuration considers predetermined factors such as the number of particles, the number of iterations, and the social and cognitive coefficients. Table 3 and Table 4 illustrate the defined configurations for PSO and the CNN, respectively.

**Figure 6:**
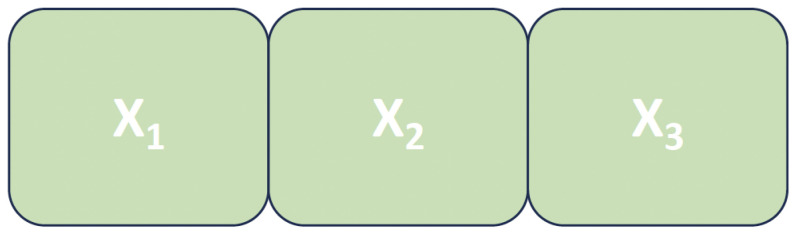
Structure of the PSOCNN approach’s particles.

The hyperparameters for optimization, including the filter size, stride, and filter number, are initialized and, then, optimized using Particle Swarm Optimization (PSO). We define a particle as having three distinct positions, each corresponding to an ideal parameter value. This particle is generated using a technique known as PSOCNN. Figure 6 illustrates the particle composition, where the position ($X_{1}$) represents the number of filters in the convolutional layer and the range is between [16, 64]. The position ($X_{2}$) represents the filter size, and [4, 8] is the search space. The third position represents the stride ($X_{3}$), which is set with the search space ranging from 2 to 4.

### 3.2. Findings and Analysis

This section presents our results and compares our approach to previous studies using two datasets: the MIAS dataset from [22] and the DDSM dataset from [22]. Furthermore, we demonstrate the optimal configurations achieved by our methodology. Given that our methodology employs a metaheuristic algorithm, executing multiple iterations of an experiment and performing a statistical analysis are necessary to attain the optimal solution.

To deploy the PSOCNN, 70% of the total images in the breast cancer database were used in the training and 30% in the testing phase. The experimental results are presented in the subsection below for each dataset.

#### 3.2.1. Analysis of PSOCNN Model for Digital Database for Screening Mammography Dataset

The following subsection presents the results of the proposed Convolutional Neural Network (CNN) model, which was trained using the hyperparameters determined by the Particle Swarm Optimization (PSO) algorithm on the DDSM dataset. Moreover, it offers a comparison with other studies and related endeavors. In addition, we demonstrate the effectiveness of Particle Swarm Optimization (PSO) in determining the best hyperparameter configurations for Convolutional Neural Networks (CNNs) to achieve interesting accuracy. This was performed by comparing the results of PSO with other methods such as a CNN [10], a YOLO-based CAD [13], an NN-based classifier [18], and a DBN-based CAD system [14]. The proposed approach achieved an accuracy of 98.23% on the test set. The CNN model developed in [10] and the proposed PSOCNN model are contrasted on the DDSM dataset in Table 5. The PSOCNN model that is proposed outperformed the CNN model without hyperparameter optimization, according to the findings shown in Table 5, whereas the most-recent one had an accuracy rate of 90.68%. The proposed PSOCNN significantly outperformed the CNN design with an accuracy of 98.23%.

Additionally, the developed PSOCNN model’s performance was contrasted with that of existing distributed research on mammography image classification utilizing the DDSM dataset shown in Table 6. These works [10,13,14,18] were chosen for comparison since they were trained using the same data. The proposed PSOCNN model is compared to previous studies in Table 6 in terms of many evaluation metrics such as the accuracy, specificity, sensitivity, precision, F1-score, and AUC. The - sign mentioned in the table indicates that the comparison approach lacks an equivalent metric. In [10], the researchers developed a CNN method for automatically classifying breast tumors using three datasets. For the DSSM dataset, the model produced an accuracy of 90.68%. In [13], the authors proposed a YOLO-CNN method for categorizing 2400 images from the DDSM dataset, achieving 97.0% accuracy, 93.20% sensitivity, and 94.00% specificity, respectively. The researchers in [14] evaluated the potential of the proposed DBN-based CAD system for the diagnosis of breast cancer using a shared digital mammography database. They used both comprehensive mass ROIs and several mass-ROI-extraction techniques. This method’s accuracy was evaluated using the DDSM dataset, and it was 92.86%. In [18], the researchers developed a novel method that involves the extraction and selection of features from multiple pre-trained CNN models, followed by classification using various machine learning algorithms: neural network (NN), k-nearest neighbors (kNN), random forest (RF), and support vector machine (SVM). This method demonstrated its superiority, particularly in terms of accuracy and sensitivity. For the DDSM dataset, an accuracy of 96% was attained. Table 6 shows that the suggested PSOCNN model performed better than the alternative classification techniques. In terms of a variety of evaluation matrices, it outperformed all comparison methods. In addition, the confusion matrix for the two classes on the DDSM dataset of the model is visualized in Figure 9.

#### 3.2.2. Analysis of PSOCNN Model for Mammographic Image Analysis Society Dataset

This section presents the results of the developed Convolutional Neural Network (CNN) model, which utilized Particle Swarm Optimization (PSO) for hyperparameter tuning, on the MIAS dataset. On the other hand, it provides a comparison with other works and comparable publications. In addition, we compared the PSOCNN to previous research to show how well PSO works at finding the best hyperparameter settings for the CNN model, which leads to much higher accuracy: CNN [9,10,11], MA-CNN [12], NN-based classifier [18]. The PSOCNN approach achieved an accuracy of 97.98% on the MIAS dataset. In addition, the performance of the PSOCNN model was compared to previous research on breast cancer detection using the MIAS dataset. The used studies for comparison [9], CNN [10], MA-CNN [12], NN-Based classifier [18], and [11], were chosen since they utilized the same dataset and were based on CNN architectures. Refs. [11,12] were both picked. As shown in Table 7, the techniques were contrasted in terms of a range of evaluation metrics such as the precision, specificity, F1-score, sensitivity, AUC, and accuracy. A missing equivalent measure is indicated in the table by the minus sign (−). In [9], the researchers created a method based on a deep belief network (DBN) for classifying mammography images. On the MIAS dataset, they yielded 91.5% accuracy, 72.4% specificity, and 94.1% sensitivity. The authors of [10] designed a CNN method for automatically classifying breast cancer using three datasets. The model’s accuracy on the MIAS dataset and DSSM dataset was 96.55%. Also in this case, the authors in [16] suggested a better CNN model that correctly categorized the MIAS breast cancer dataset with 89.47% sensitivity, 90.71% specificity, and 90.50% accuracy. In [18], the researchers developed a novel method, as explained in Section 3.2.1. They achieved an impressive accuracy of 94.5% on the MIAS dataset. In addition, in [11], the researchers developed a new technique for the CAD of unusual breasts in mammography images by combining three components: SVM, WFRFT, and PCA. In the instance of SVM, this resulted in a sensitivity of 92.22%, a specificity of 92.10%, and an accuracy of 92.16%. For classifying the mammography images. Referring to Table 7, the proposed PSOCNN technique outperformed the alternatives. In terms of all evaluation metrics, it exceeded all comparison approaches.

Since our method uses a metaheuristic algorithm, statistical analysis and numerous iterations of the experiment are needed to obtain results and study convergence to the best outcome.

### 3.3. Discussion

One of the most-difficult issues in breast cancer diagnosis is distinguishing between malignant and nonmalignant patients. This work contributes by classifying mammography datasets by building a CNN architecture from scratch and evaluating its capacity to classify benign and malignant cases using the CNN hyperparameter optimization technique. As previously stated, the results of the comparison of the PSO algorithm with other optimization algorithms revealed that the PSO efficiently selects the optimal hyperparameter values for the CNN architecture to achieve high accuracy. It was contrasted with [9,10,11,12,13,14]. Table 6 and Table 7 present a comparison between the proposed PSOCNN model and other competing models across different datasets. According to the compared results, PSO was more suitable for working with the CNN architecture to classify breast imaging data. The optimal values for the CNN hyperparameters were successfully determined, leading to the maximum accuracy achieved.

**Figure 7:**
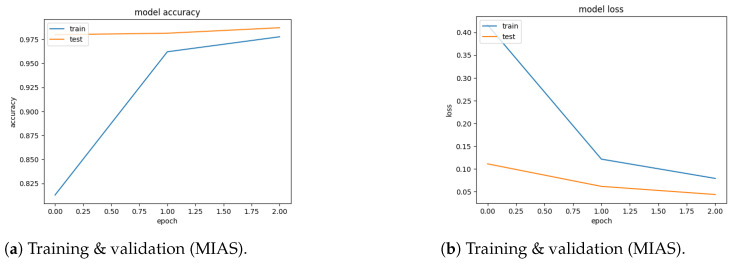
(**a**) Accuracy (**b**) Loss.

**Figure 8:**
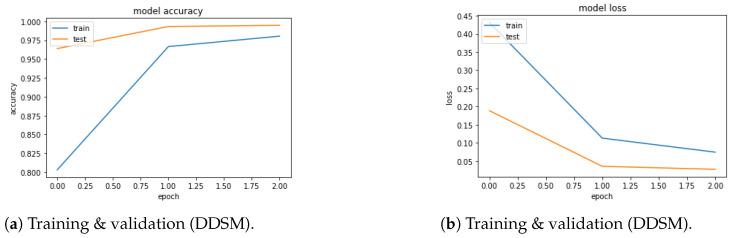
(**a**) Accuracy (**b**) Loss.

**Figure 9:**
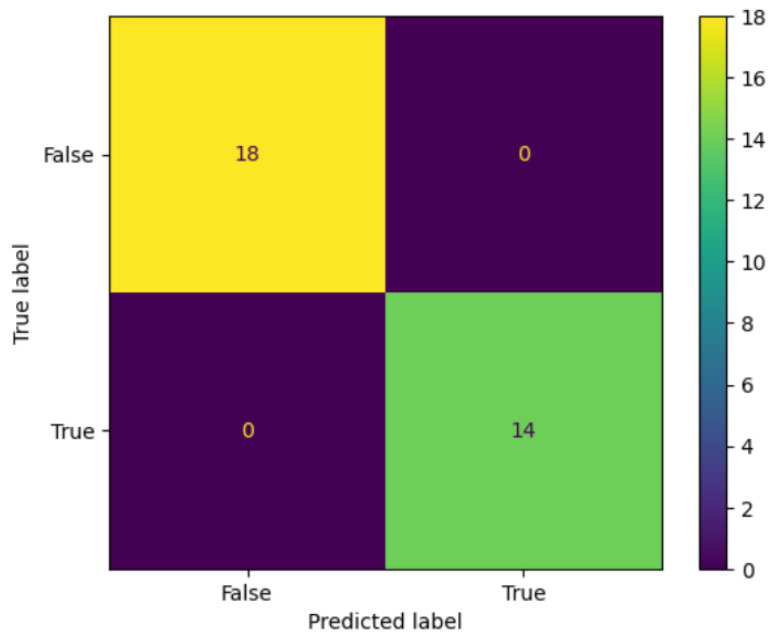
The confusion matrix for the two classes on the DDSM dataset.

Finally, the following outcomes from the existing experiments are interesting:

- According to the accuracy, the suggested PSOCNN model outperformed the other models, indicating that the PSO outperformed the other methods when the PSO optimized new hyperparameters. The PSOCNN model reached 98.23% accuracy for the DDSM dataset and 97.98% accuracy for the MIAS dataset. Furthermore, the PSOCNN model beat all other examined models.
- Figure 7a and Figure 8a show the training curves of the optimal model, as determined by PSOCNN, on the DSSM and MIAS datasets. These figures allowed us to evaluate the performance of the CNN model generated by our proposed method. The graph of the learning curves displays a good fit. In Figure 7b and Figure 8b, the training loss curve exhibits a gradual decrease until it reaches a state of stability. As the validation loss curve reaches a state of stability, it becomes distinct from the training loss curve with a small gap.
- The optimal solution detection is one of our most-significant achievements, which is supported by the confusion matrix in Figure 9.

#### Limits of the PSOCNN

This research presented a significant breast cancer classification technique that uses a customizable Convolutional Neural Network (CNN) architecture and a metaheuristic optimization algorithm. Future research should investigate other limitations, even if the suggested PSOCNN model provides great classification performance in the classification of mammography pictures. The following examples demonstrate the constraints of the PSO algorithm and the proposed PSOCNN models:

The PSOCNN was exclusively used to classify mammography datasets. These results may not generalize to other datasets because they are limited to the MIAS dataset and the DDSM dataset.

The PSO technique is only successful in detecting the hyperparameter values of the CNN architecture, and we cannot generalize it to other pre-trained CNN designs.

PSO has hyperparameters (such as inertia weight and acceleration coefficients) that require careful adjustment. The selection of these hyperparameters can have an impact on PSOCNN’s performance. Dependence on initialization: The initializing positions of particles can impact the performance of PSO. Random initialization can produce a variety of solutions, and determining a suitable initializing technique can be difficult.

When using the PSOCNN, researchers must often carefully analyze the characteristics of their specific task, dataset, and neural network design to achieve successful and robust optimization.

## 4. Conclusions and Future Challenges

DL is one of the key methods for classifying medical images. Layerwise automatic feature extraction is a typical characteristic of DL methods used for biomedical image classification, such as Convolutional Neural Networks. Knowing how to tune hyperparameters is necessary for preparing CNNs for classification goals. Each layer has its own set of hyperparameters. To obtain exceptional results, these hyperparameters must be adjusted because they affect how well a CNN model performs. Choosing hyperparameters is not a good idea because it is a time-consuming and challenging task. In several disciplines, hyperparameter optimization has been profoundly influenced by metaheuristic methods. This paper proposed a new breast cancer classification technique based on the CNN architecture and an optimization algorithm. The most-popular optimization approach is called Particle Swarm Optimization (PSO). The hyperparameters of the CNN architecture are optimized using the swarm optimization (PSO) technique, which results in the PSOCNN model. The four stages of this PSOCNN model are: (1) developing the CNN’s architecture; (2) optimizing hyperparameters; (3) learning; and (4) performance evaluation. Different studies and CNN methodologies were contrasted with the PSOCNN. The comparison results showed how well the proposed approach detected breast cancer. The evaluation utilized two datasets: DDSM and MIAS. We compared the PSOCNN model to other models to show how useful PSO is for finding the best CNN model hyperparameter settings that can obtain good results. The experiment results validated the importance of the suggested method. Accuracy, specificity, sensitivity, F-score, and precision were used as the metrics for measuring the performance of the recommended algorithm. The results showed that our proposed algorithm performed better than the other competitive algorithms, indicating that it is possible to improve the performance of classification models for breast cancer diagnostics by utilizing PSO as a metaheuristic algorithm of optimization to select the CNN hyperparameters. Future research will assess the suggested model using multiple datasets with additional images. Along with the suggested PSO technique, pre-trained models like DensNet201, ResNet, Inception, and DensNet121 will be utilized for breast cancer detection. For hyperparameter adjustment, multiple metaheuristic techniques will also be used. Additionally, we will evaluate the performance of the suggested approach in dealing with various medical image classification challenges and diagnostic applications, utilizing the PSO method to handle further medical and other issues. The classification accuracy will be further increased by combining the CNN architecture with a variety of feature-extraction techniques. The effectiveness may be improved through transfer learning with other models. In the future, studies will examine the use of PSO to optimize other deep learning parameters, such as activation functions, epochs, and a number of convolutional layers. As a result, additional optimization approaches and CNN designs must be tested to assess the efficacy of computational cost and complexity.

## Figures and Tables

**Table 1:** Mammogram dataset specifications used in the classification stage.

Database	Number of Benign Pictures	Number of Malignant Pictures	Number of Total Pictures
DDSM	5970	7158	13,128
MIAS	2376	1440	3816

**Table 2:** CNN hyperparameters.

Hyperparameters	Description
Kernel (filter) size	Kernel size in the convolutional layer
Feature map number	Size of the convolutional layer’s kernel
Stride	Number of pixels moved within the kernel during convolution
Padding	Hyperparameters to obtain the boundary region features of the training data
Pooling type	Value calculation of each feature patch (average, maximum)
Number of epochs	Number of iterations
Number of layers	Number of layers that make up the entire network
Number of neurons	Number of neurons in the fully connected layer
Batch size	Group size is used to divide the training data into multiple groups
Weight initialization	Weight initialization with a small random number (Xavier initialization, He initialization)
Loss function	This function calculates the error including the cross-entropy and MSE
Optimizer	The argument needed to compile the model (SGD, Adam, RMSprop, Adadelta, etc.)
Dropout rate	Depending on the desired probability, the algorithm removes units from the neural network
Activation function	Activation function of neurons (ReLU, Sigmoid, etc.)

**Table 3:** CNN static parameters.

CNN Static Parameters	
Activation function (classifying layer)	Sigmoid
The function of nonlinearity activation	ReLU

**Table 4:** PSO static parameters.

PSO Static Parameters	
Particles	4
Iterations	3
Cognitive weight (W)	2
Social constant (W2	2

**Table 5:** Comparison between the proposed PSOCNN and CNN models on the DDSM dataset.

Model	Dataset	Accuracy
CNN [10]	DDSM	90.68%
Our proposed method (PSOCNN)	DDSM	98.23%
Improvement (%)	-	8%

**Table 6:** Comparison between the proposed PSOCNN model and the related works on the DDSM dataset.

Model	Dataset	Accuracy	Sensitivity	Specificity
CNN [10]	DDSM	90.68%	-	-
YOLO-based CAD [13]	DDSM	97%	93.20%	94.00%
NN-based classifier [18]	DDSM	96%	94.70%	-
DBN-based CAD system [14]	DDSM	92.86%	-	-
Our proposed method (PSOCNN)	DDSM	98.23%	-	-

**Table 7:** Comparison of the developed PSOCNN model against other relevant studies on the MIAS dataset.

Model	Dataset	Accuracy	Sensitivity	Specificity
DBN [9]	MIAS	91.5%	-	-
CNN [10]	MIAS	96.55%	93.20%	94.00%
MA-CNN [12]	MIAS	96.47%	-	-
[16]	MIAS	90.50%	90.71%	-
WFRFT + PCA + SVM [11]	MIAS	92.16%.	92.10%	-
NN-based classifier [18]	MIAS	94.5%	96.32%	-
Our proposed method (PSOCNN)	MIAS	97.98%	-	-

## Data Availability

The data presented in this study are openly available in [22].

## Abbreviations

The following abbreviations are used in this manuscript:

CNN	Convolutional Neural Network
PSO	Particle Swarm Optimization
PSOCNN	Particle Swarm Optimization Convolutional Neural Network
DL	Deep learning
MIAS	Mammogram Image Analysis Society
DDSM	Digital Dataset for Screening Mammography
